# Atypical Power Doppler Ultrasound Findings in Juvenile Idiopathic Inflammatory Myositis (JIIM) Flare

**DOI:** 10.7759/cureus.14949

**Published:** 2021-05-11

**Authors:** Brandon Ewing, Richard Thomas, Hannah Elsinghorst, Rabheh Abdul-Aziz

**Affiliations:** 1 Pediatric Rheumatology, Jacobs School of Medicine and Biomedical Sciences, Buffalo, USA; 2 Radiology, Jacobs School of Medicine and Biomedical Sciences, Buffalo, USA

**Keywords:** myositis flare, gray scale ultrasound, juvenile idiopathic inflammatory myositis, power doppler ultrasound, dermatomyositis

## Abstract

Juvenile idiopathic inflammatory myositis (JIIM) is a multisystem inflammatory disease that impacts the muscles, skin, and blood vessels. Gray-scale power Doppler ultrasound is a technique that can be used to assist the diagnosis of JIIM and myositis in general. We report a case of an atypical symptomatic JIIM myositis flare that shows increased muscle echogenicity without the corresponding increase (complete absence) of Doppler flow.

## Introduction

Juvenile idiopathic inflammatory myositis (JIIM) is a multisystem inflammatory disease that impacts the muscles, skin, and blood vessels [1]. Major symptoms include rashes, pain, and weakness of the arms and legs causing difficulty in walking, climbing stairs, and lifting objects above the head [2]. Diagnosis of JIIM and accompanying disease flares are traditionally based on the Bohan and Peter criteria [2]. Definite juvenile dermatomyositis consists of classic skin involvement and at least three of the following: 1) proximal muscle weakness, 2) elevation of a muscle enzyme(s), 3) myopathic changes on electromyography, and 4) abnormal muscle biopsy suggestive of inflammatory myopathy [3,4]. Probable juvenile dermatomyositis (JDM) is defined as patients who have the characteristic rash and fulfil only two of the above criteria. An expanded definition was proposed in 2006 using an international consensus survey [5]. These new criteria include: 1) typical findings on muscle magnetic resonance imaging (MRI), 2) nailfold capillaroscopy abnormalities, 3) calcinosis, and 4) dysphonia [5]. Imaging techniques, such as MRI, are often heavily used in cases where the diagnosis is equivocal or where reporting of symptomology may be unreliable, such as when treating young children who cannot express or explain symptoms; however, MRI remains expensive and cumbersome and has a high false-negative rate [6,7]. Gray-scale ultrasound with power Doppler is a modality that been postulated to be a potential alternative for diagnosis that is not only less expensive than MRI, but also highly sensitive to detecting myositis patterns found in JIIM [8].

## Case presentation

**Figure 1:**
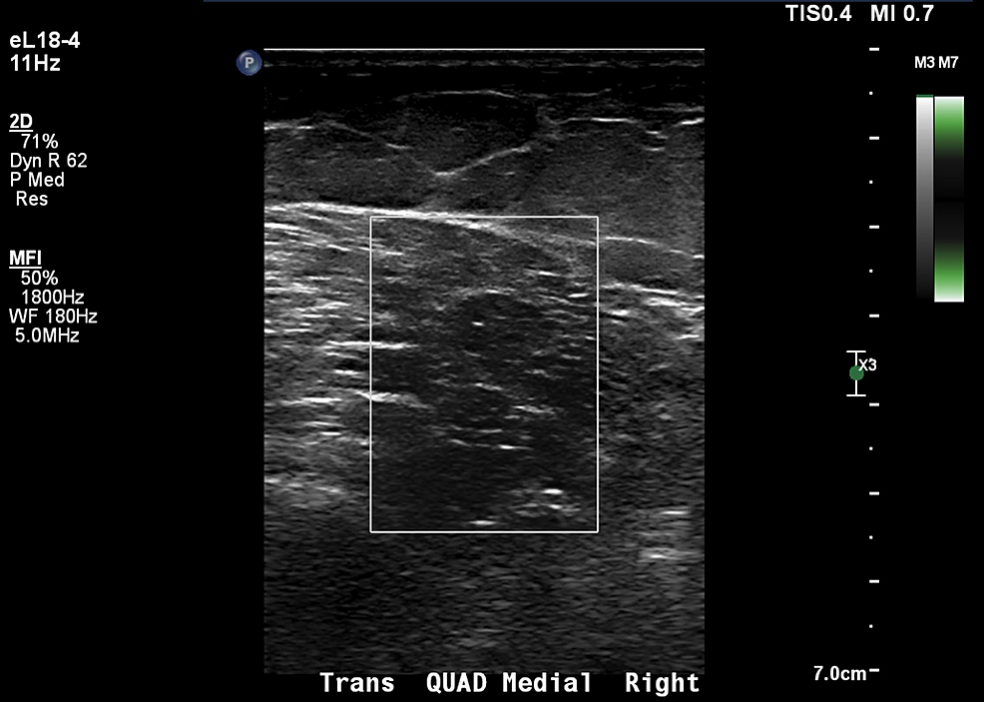
Gray-scale power Doppler ultrasound: transaxial view of the quadriceps muscle (square) showing absent Doppler flow in the context of active myositis flare

A 17-year-old male presented with a two-year history of skin rashes over his hand and lower extremities weakness. Physical examination revealed Gottron changes on his right third metacarpophalangeal joint and proximal interphalangeal joints, right fourth proximal interphalangeal joints, and left fifth metacarpophalangeal joint and proximal interphalangeal joints. A patch of dry skin with mild erythema was discovered on the left ankle and on the anterior aspect of the lower leg. Laboratory workup showed elevated muscle enzymes, including elevated aspartate aminotransferase (186 IU/L), alanine aminotransferase (341 IU/L), aldolase (17.8 U/L), LDH (350 U/L), and CK (1,092 U/L). Based on modified Bohan and Peter criteria, he was diagnosed with dermatomyositis [5]. High resolution and frequency (Res) gray-scale static and cine imaging with power Doppler, along with an MRI, were performed to assist with the diagnostic workup. The MRI showed findings congruent with a typical flare of JIIM. Lower extremity ultrasound images with corresponding ultrasound parameters depicted below, however, showed increased muscle echogenicity consistent with dermatomyositis, but with no detectable Doppler flow (Figure 1).

**Figure 2:**
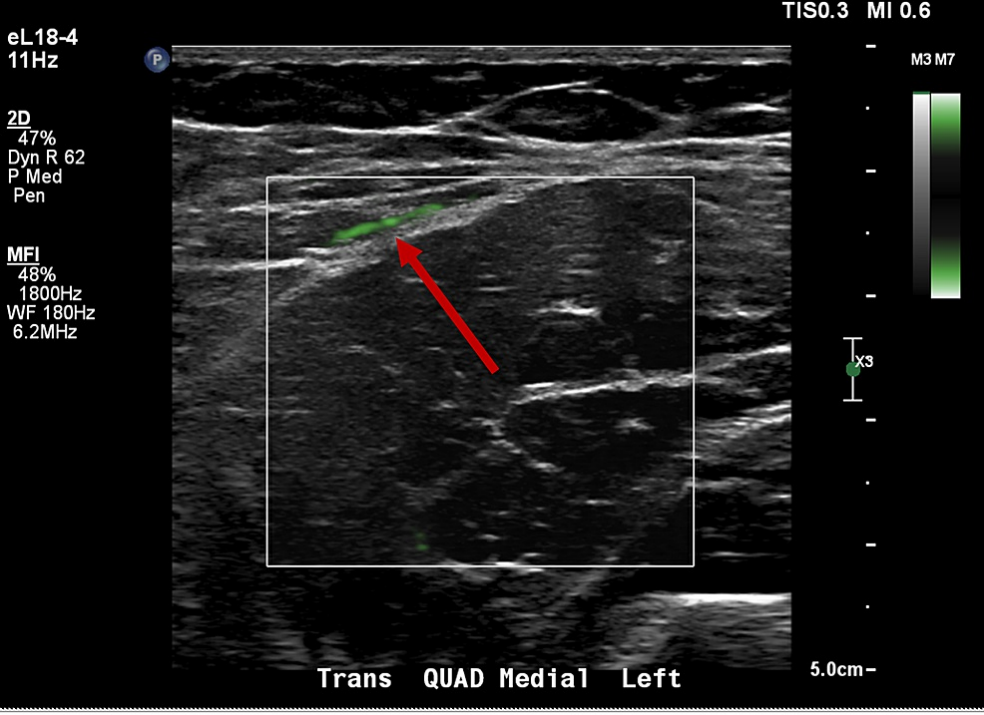
Power Doppler ultrasound: transaxial view of the quadriceps muscle (square) showing typical Doppler pattern (green) in cases of active myositis flare

Typically, increased muscle echogenicity in inflammatory processes coincides with mild to moderate increases in Doppler flow unless a pathological process, such as compartment syndrome, is occurring [8]. In this case, compartment syndrome was clinically absent. To our knowledge, the pairing of this finding of increased muscle echogenicity with complete absence of Doppler flow has not been documented in a patient with JIIM flare. An example showing the typical power Doppler ultrasound findings in JIIM, along with corresponding parameters, has been provided (Figure 2).

## Discussion

The lack of discrimination of myositis is important to both the research community and the practicing clinician. Consensus on the utility of gray-scale power Doppler ultrasound in the workup of JIIM and other inflammatory myopathies remains limited but evolving. Early clinical studies indicated that power Doppler ultrasound, in conjunction with gray-scale ultrasound, could reasonably be used in the diagnosis of myositis-related diseases, such as JIIM. [8]. Specifically, Meng et al. found a statistically significant difference in peak vascularity when comparing inflamed muscle to non-inflamed muscle [9]. Later studies, however, have focused on attempts to standardize reporting of power Doppler ultrasound findings in JIIM and other myositis related diseases [8]. A study conducted by Weber et al. was able to demonstrate the increased perfusion of muscle during symptomatic myositis flare on power Doppler ultrasound by analyzing the replenishment kinetic of microbubbles, perfusion-related parameters, and flow volume and velocity [10]. It was noted that contrast-enhanced blood flow had the greatest sensitivity, specificity, positive predictive value (PPV), and negative predictive value (NPV) for the diagnosis of dermatomyositis and polymyositis [10]. There have been no recent trials that examine the true sensitivity and specificity of gray-scale power Doppler ultrasound in the diagnosis of JIIM or related flares.

## Conclusions

Based on the symptomatology and MRI findings, our patient was experiencing a JIIM flare; however, the power Doppler ultrasound findings were more suggestive of compartment syndrome, a pathological condition that we know was not present in this patient. Our case provides an example of how ultrasound images with Doppler can be misleading in JIIM and may not fully detect a flareup. More research is needed to understand the diagnostic potential of gray-scale ultrasound with power Doppler as well as its relative sensitivity and specificity in the diagnosis of JIIM and other related diseases.
